# Sleepiness, Long Distance Commuting and Night Work as Predictors of Driving Performance

**DOI:** 10.1371/journal.pone.0045856

**Published:** 2012-09-21

**Authors:** Lee Di Milia, Naomi L. Rogers, Torbjörn Åkerstedt

**Affiliations:** 1 The Institute of Health and Social Science Research, School of Management, Central Queensland University, Rockhampton, Queensland, Australia; 2 Chronobiology and Sleep Unit, and Institute of Health and Social Science Research Central Queensland University, Rockhampton, Queensland, Australia; 3 Stress Research Institute, Stockholm University, Stockholm, Sweden; University of Alabama at Birmingham, United States of America

## Abstract

Few studies have examined the effect of working night shift and long distance commuting. We examined the association between several sleep related and demographic variables, commuting distance, night work and use of mobile phones on driving performance. We used a prospective design to recruit participants and conducted a telephone survey (n = 649). The survey collected demographic and journey details, work and sleep history and driving performance concerning the day the participant was recruited. Participants also completed the Karolinska Sleepiness Scale and the Epworth Sleepiness Scale. Night workers reported significantly more sleepiness, shorter sleep duration and commuting longer distances. Seven variables were significant predictors of lane crossing. The strongest predictor was acute sleepiness (OR = 5.25, CI, 1.42–19.49, p<0.01) followed by driving ≥150 kms (OR = 3.61, CI, 1.66–7.81, p<0.001), obtaining less than 10 hours sleep in the previous 48 hours (OR = 2.58, CI, 1.03–6.46, p<0.05), driving after night shift (OR = 2.19, CI, 1.24–3.88, p<0.001), being <43 years old (OR = 1.95, CI, 1.11–3.41, p<0.05) and using mobile phones during the journey (OR = 1.90, CI, 1.10–3.27, p<0.05). Sleep related variables, long-distance commuting and night work have a major impact on lane crossing. Several interventions should be considered to reduce the level of sleepiness in night workers.

## Introduction

There is substantial evidence implicating the contribution of driver sleepiness in motor vehicle crashes. Estimates suggest sleepiness may explain up to 23% of crashes [1]–[3] and furthermore, long distance highway drivers may face a greater risk [4]–[6]. The extent of driver sleepiness appears widespread and is found in several countries. A representative survey of North Americans found 6% drive sleepy at least three times per week and 37% drive sleepy at least once per month [7]. Two-percent of French drivers (n = 35,000) reported weekly bouts of sleepiness so severe it required them to stop driving and 9% reported this experience each month [6]. In Norway, approximately 8% of drivers reported falling asleep while driving in the previous year [8].

Sleepiness may result from several factors including the interplay between circadian and homeostatic processes [9], sleep disorders [6] and characteristics associated with the journey such as time at the wheel and time of day [3], [4], [10], [11]. These factors do not act independently and often combine to exert their influence. For example, Sagaspe et al. [12] demonstrated that a long distance drive from 9 pm to 5 am resulted in a greater number of errors than a two-hour drive undertaken during the circadian nadir.

Work schedules that involve night work also result in sleepiness [13], [14]. The main reason for night shift sleepiness is the amount of prior wakefulness and working during the circadian nadir [15]. Another factor that contributes to increased sleepiness is that night workers obtain insufficient sleep during the day [16] and this increases the pressure to sleep. In particular, sleepiness has been shown to increase across the night shift [17], [18] and thus, night workers have higher levels of sleepiness prior to commuting. Epidemiological [3], [11], simulator [19], [20] and survey studies [21]–[23] have linked driving after night shift with a greater crash risk. Stutts et al., [11] concluded the odds ratio for crashes were highest among drivers that worked at night, slept less than 6 h per day, had greater wakefulness and worked more than 60 h per week. Åkerstedt et al. [19] compared the driving performance of the same participants when fully rested and after night shift. Driving after night shift resulted in a four-fold increase in driving accidents and greater lane drift. In addition, subjective sleepiness was highest following 49 minutes of driving compared with 113 in the rested condition. Survey studies report driving after 12 h night shifts, short sleep durations and commuting more than 35 minutes resulted in greater sleepiness, and impaired driving performance [21].

There do not appear to be studies that have directly examined the impact of long distance commuting on driving performance following night work. It is reasonable to expect longer commutes would increase the driving risk given the driver is already sleepy from the night shift and driving performance is known to decay with journey time [6], [20]. Some evidence for the impact of long distance commuting comes from a cross-over study that required participants to drive 200 kms at 09∶00 following a full night sleep and after being restricted to 2 h sleep ending at 01∶00 [10]. Performance following sleep restriction resulted in an eight-fold increase in the number of lane crossings. In a survey of long distance commuters (M = 212 kms) 1% of drivers with a full night reported severe sleepiness (≥7 Karolinska Sleepiness Scale, KSS) compared with 19% that had worked night shift [24].

The aim of this study is to address a gap in the literature concerning sleepiness, long distance commuting, night work and performance. Thus the study has implications for the safety of commuters following night work. We propose higher levels of sleepiness and longer distances will result in impaired driving performance. The present study was conducted in rural Australia; a location where the relative risk of a fatigue related crash is 13 times greater compared with an urban region [25], [26]. The primary explanation for the higher crash risk appears to be due to human factors.

## Methods

### Ethics Statement

The study protocol was approved by the Human Research Ethics Committee at Central Queensland University. Participants were provided with an information sheet and written consent was obtained from those agreeing to take part in a telephone survey. Agreeing to participate in the study was not however binding and participants were free to discontinue participation at any time and have their data deleted.

### Participants

We conducted the study in Central Queensland (Australia). Long distance commuting is a feature of this rural region given the distances between local towns and larger regional centres, and employment in the mining sector. For lifestyle reasons some workers choose to live on the coast and commute to work. One estimate suggested 11% of the workforce regularly commutes long distances before and after work [24].

We took advantage of a planned operation by the Queensland Police Service to conduct random breath tests (RBT) to recruit participants. Recruitment took place on three regional highways between 08∶00 and 10∶00 over a two-week period (Week 1; Monday-Tuesday, Week 2; Wednesday-Friday) in November 2010. Non-commercial drivers travelling in an easterly direction were randomly stopped. Following the RBT drivers were told they were free to leave but could choose to participate in a survey on driving habits. Interested drivers were directed to interviewers positioned approximately 50 m away.

Interviewers identified themselves as University employees and emphasised the study was not connected in any way with the RBT. Drivers were informed that participation was voluntary, confidential and data would be collected via a telephone interview. All drivers were asked for their age and gender, and those interested in further participation provided written consent, a preferred contact time and telephone number. Participants completing the survey were eligible to win one of fifteen A$100 vouchers.

### Data Collection and Analysis

Participants completed a number of scales and in this study we report on the following data:

1. Demographic variables including work history and distance travelled. Age was divided into <43 and ≥43 years; distance (<150 kms, ≥150 kms); worked night shift (yes/no); weekly working hours (≤40, >40). The age split was based on the median age for the sample. Ting et al. (2008) suggested driving performance deteriorates after 80–90 minutes of driving and given the road speed in this region, 150 kms was used to indicate a ‘safe’ driving distance. Participants that worked night shift were assigned into a NW group and the others were classified as NNW (no night work).
2. Participants reported whether they had ever been diagnosed with a sleep disorder (yes/no), whether they snored (yes/no), the amount of sleep obtained in the previous 24 and 48 hours (≤5, ≥5 per 24 h), and the number of nights with full sleep in the past week (≤4, >4) [3]. A full night’s sleep was defined as “something like 7 h or more of sleep taken between 10 pm and 6 am.” [3].
3. The KSS [27] was used to record sleepiness at the RBT site (Time1) and at the end of the commute (Time2). Values ≥7 were used to categorise severe sleepiness [24].
4. Chronic sleepiness was categorised as values ≥10 [3], [28] using the Epworth Sleepiness Scale (ESS [29]).
5. Whether they used a mobile phone or similar device during the journey (yes/no).
6. Participants reported whether they fell asleep during the journey (yes/no). Driving performance was assessed in terms of a single or multiple vehicle crash (or object), or crossed the centre line/outside edge of the highway. The number of reported crashes or lane crossing were categorised as yes/no.

The majority of telephone interviews (78%) were completed within three days of recruitment and the balance were completed within a week. The survey took approximately 28 minutes to complete. We used a multivariate logistic regression model to assess the relationship between the predictor variables and driving performance. The predictor variables were entered simultaneously in order to derive the adjusted odds ratio (OR).

## Results

**Figure 1:**
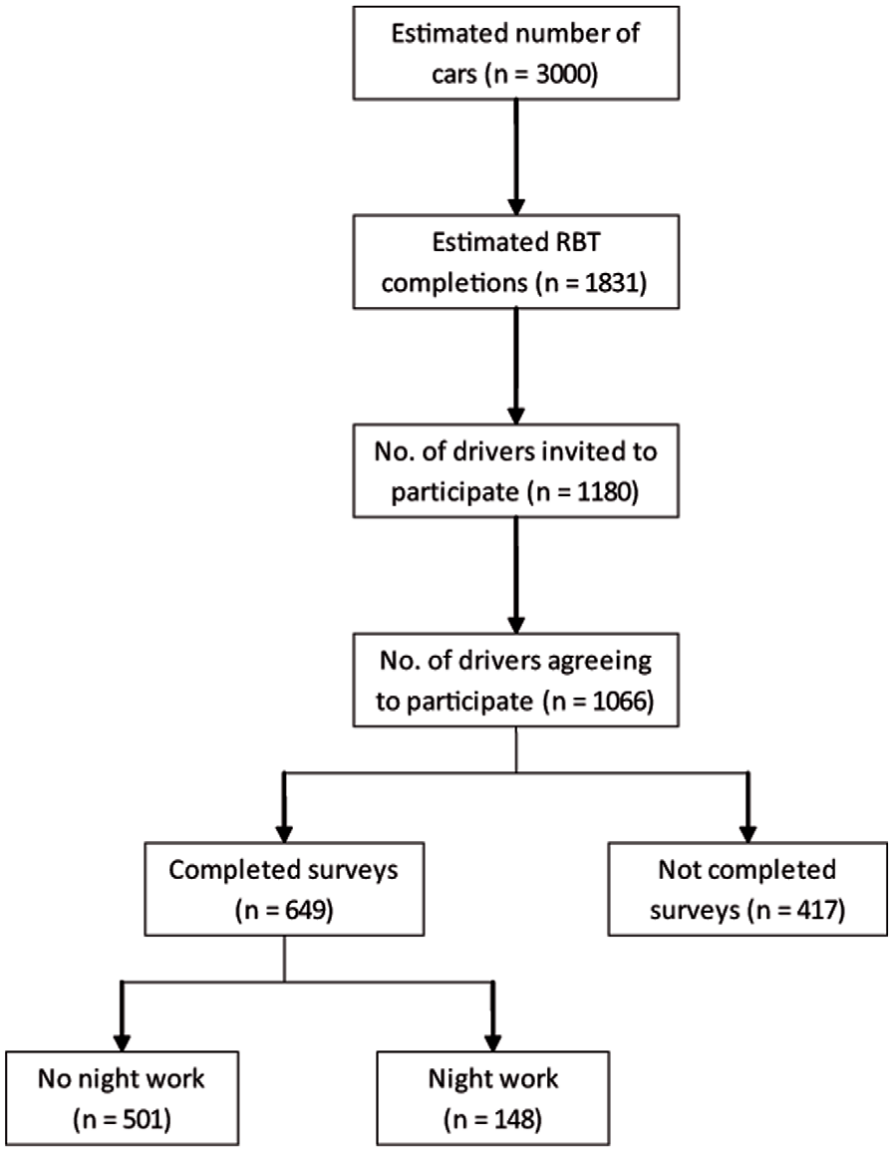
Flow chart showing participation in the study.

A breakdown showing the maximum number of drivers on the highways and the final survey respondents is shown in Figure 1. One-thousand and sixty-six drivers agreed to be contacted and we interviewed 61% of these drivers (n = 649). The main reason for not participating was our inability to contact the participant after three attempts (25%) and 8% declined to participate.

The respondent’s mean age was 43.83±13.46 years and 486 were male (75%). The mean age of the non-responders (n = 417) was 41.68±13.51 years and 72% were male. Based on these characteristics the groups appeared similar. The NW group comprised of 148 drivers (23% of the sample). The mean age was 40.67 years (±11.64) and 140 were male. The NNW group were on average 44.77 years old (±13.82) and 346 were male. The NW group worked a mean of 11.7 h (±2.87) prior to their commute.

A multivariate ANOVA compared the two groups. The data in Table 1 indicate the NW group commuted significantly longer distances, experienced greater sleepiness and obtained less sleep compared to the NNW group. No differences on the ESS were observed.

**Table 1:** Descriptive statistics for distance travelled, sleepiness and sleep duration for night workers (NW) and non-night workers (NNW).

	NW		NNW		
	Mean	SD	Mean	SD	p
Distance travelled at Time 1 (kms)	140.29	72.17	117.55	89.74	.001
Total distance (kms)	229.62	125.86	182.79	139.76	.001
Sleepiness (KSS) at Time 1	3.10	1.87	1.99	1.42	.001
Sleepiness (KSS) at Time 2	3.51	1.86	2.00	1.39	.001
Sleep in previous 24 h	6.82	2.21	7.74	1.36	.001
Sleep in previous 48 h*	6.94	1.90	7.62	1.35	.001
Number of nights with fullsleep	3.93	2.21	5.43	2.21	.001

* shown per 24 h period.

Seven drivers (5 NW) fell asleep at least once during their commute and six, reported falling asleep before reaching the RBT site. No crashes were reported but 90 lane crossings were reported. The NW group were involved in significantly more lane crossings (χ^2^ = 33.80, p<0.001). Table 2 provides the adjusted OR for the variables associated with lane crossings. Seven of the 12 predictor variables were significant and three of these variables were sleep related. The variables with the strongest association with lane crossings were excessive sleepiness (KSS≥7), driving more than ≥150 kms and driving after the night shift. Sleep disorders and chronic ESS were not significant predictors. Chi-square test found no significant differences for sleep disorders between the NW and NNW groups.

**Table 2:** Adjusted odds ratio for variables associated with lane crossing.

Variable	Category	Adjusted OR*	95% CI	p
Acute sleepiness (KSS)	<7	1		
	≥7	5.25	1.42–19.49	.01
Distance driven	<150 kms	1		
	≥150 kms	3.61	1.66–7.81	.001
Sleep in previous 48 h**	≥10	1		
	<10	2.58	1.03–6.46	.05
Worked night	No	1		
	Yes	2.19	1.24–3.88	.001
Snore	No	1		
	Yes	2.00	1.10–3.66	.05
Age	≥43 years	1		
	<43 years	1.95	1.11–3.41	.05
Using mobile	No	1		
	Yes	1.90	1.10–3.27	.05
Weekly work hours	≤40	1		
	>40	0.65	.36–1.19	.16
Sleep disorder	No	1		
	Yes	1.25	.40–3.80	.70
Chronic sleepiness(ESS)	<10	1		
	≥10	1.44	.77–2.71	.26
Sleep in previous24 h	≥5	1		
	<5	1.04	.44–2.38	.93
Number of nightswith full sleep	>4	1		
	≤4	1.50	.84–2.67	.17

* variables were entered simultaneously into the model.

** shown per 24 h.

## Discussion

The present study addressed a gap in the literature concerning long distance driving following night work. We proposed that higher levels of sleepiness, long distance commuting and NW would be associated with impaired driving performance. Our results supported this argument. The NW group reported higher levels of sleepiness during – and at the end of the commute, had taken less sleep across the last week and commuted longer distances than the NNW group.

Consistent with previous studies we found sleep-related variables [1]–[3], [6], [10], [11], [19] and in particular, acute sleepiness, to be the strongest predictors of impaired driving performance [3], [4]. Drivers with acute sleepiness were five times more likely to report lane crossing and those with less than five hours of sleep per day over the previous two days reported a three times greater risk of lane crossing [3], [4], [12]. It is important to highlight that these effects were independent of each other. Consistent with another prospective study we found no evidence for chronic sleepiness as a risk factor [3]. This may be because the ESS is less sensitive when used in prospective designs that aim to link present sleepiness and outcomes. In contrast, the ESS has been associated with crash involvement in retrospective designs [6].

Where other studies have found a link between sleep disorders and crashes [6] we did not find this relationship. This may be because drivers were asked whether they had ever been diagnosed with a sleep disorder rather than did they have a present diagnosis. Many participants reported snoring and this variable was associated with driving performance (OR = 2.00; 95% CI, 1.10–3.66, *p*<.01). Snoring and obesity is a symptom typically associated with sleep apnea and given the sample was mostly male and aged in their forties, it may be that some participants had an undiagnosed sleep disorder. Some 24% of males aged 40–49 are estimated to have sleep apnea [30] and a large study of North American police officers found over 40% screened positive for an undiagnosed sleep disorder [31]. As a post-hoc analysis we found that those reporting snoring were significantly (χ^2^ = 25.16, p<0.001) more likely to be obese (body mass index ≥30 km/m^2^).

The sleep duration data were self reported and therefore we cannot confirm their accuracy. The reported mean sleep for the NW group is consistent with a range of 6.4 h and 7.2 h obtained from an Australian study in a similar region [32] and mean sleep of 6.6 h reported for workers in the North Sea [17]. Nonetheless, the sleep estimates are almost an hour less than the estimates from the NNW group. This may suggest chronic sleep loss in the NW group from an accumulation of sleep debt across successive night shifts.

Long distance driving was the second main predictor of lane crossing and the result is in line with the literature. In a case-control study Cummings et al. [4] reported crash risk increased by a factor of 2.2 for every 100 miles driven and simulated driving studies suggest that driving performance decays with time at the wheel over a 90-minute period [25].

Working the night shift increased the risk of impaired driving performance and epidemiological studies have reported this relationship [3], [11]. However, a weakness of these studies is they do not necessarily confirm that the temporal relationship between the crash and night shift; rather that the variables are linked. In contrast, our study directly addresses the temporal relationship between ending night work and driving performance.

The strengths of this study include the use of a prospective field design that recruited drivers across three regional highways. The interviews were mostly completed within three days of identifying drivers and this reduces the possibility of recall bias. Finally, we employed established scales and obtained a good response rate to the survey.

At the same time there are some limitations to the study. The data are self-reported and this raises the possibility of some bias. It is well known that shift workers trade off health and safety for other benefits associated with long work hours [33]. Shift workers receive a good deal of fatigue management training that outlines the importance of sleep [34] and therefore the sleep estimates may be over estimated. However, even if this bias is present the sleep reported by night workers is less than that of non-night workers. A second limitation may be whether the Police presence at recruitment led to bias. We do not think this is a source of bias. First, we emphasised to participants the independence of the study. Second, some 35% of drivers that participated in the random breath test were not interviewed by the road-side research assistants (see Figure 1). Another limitation is whether we surveyed a representative sample. Data is not available to identify the characteristics of road use by time of day. However, we demonstrated that within the study the respondents and non-respondents were similar in terms of age and gender. Furthermore, age, gender and commuting distances found in this study were similar to those obtained in an earlier study in the same region [24]. Finally, we checked the traffic volume for November and it appeared to be consistent with any other non-holiday month. A fourth limitation is using lane crossing as our indicator of driving performance. Lane crossing is a precursor to a crash and its sensitivity as a measure of driving performance has been established in several simulator and field studies [6], [10], [12], [19]. One study found that drivers reporting one near-miss crash were 1.2 times more likely to report at least one real crash [35]. We agree that objective driving performance is desirable but this is difficult to achieve in a population prospective design. Nonetheless, future studies should aim to collect objective indicators of sleepiness and driving performance.

The findings in this study suggest the need for countermeasures to reduce the number of lane crossing episodes. We focus on NW given this group reported the greatest amount of sleep loss and the longest commutes. At the organizational level there are two interventions that may reduce the level of sleepiness. Napping is one strategy to reduce the amount of wakefulness and one study examined the benefits of taking a 20-minute nap taken at 03∶00 [36]. The results suggested a significant improvement in reaction time but not for sleepiness during the home commute. Future studies could examine nap duration and timing on subsequent alertness. Wakefulness may also be reduced by ending the last night shift early to allow some sleep before the commute. Another organisational intervention is to trial the use of bright light during the night shift [37]. At the individual level drivers could take frequent driving breaks [4], [10] but while many drivers recognise sleepiness symptoms few will stop driving [2]. Another countermeasure is the use of drug treatment to reduce sleepiness. Randomised control studies have reported 200 mg of modafinil reduced by one-half the number of accidents and near accidents reported during the morning commute [38] and armodafinil was also reported to reduce sleepiness during the night shift and the home commute [39]. The refinement of crash avoidance technologies such as lane departure warning systems may also assist to alert sleepy drivers of impending risk [40]. Legal enforcement may also assist to reduce the prevalence of driving sleepy. The development of a short but valid road-side sleepiness ‘test’ may assist Police to identify sleepy drivers [41]. This technology may also be used by drivers as a self-assessment tool to guide their decision to drive or rest. Collectively, these interventions may assist to promote a reduction in driver sleepiness.

### Conclusions

Night workers commuting after work were found to have increased levels of sleepiness, had less sleep in the previous week and travelled a longer distance in comparison to non-night workers. Night workers were more likely to report lane crossing. The adjusted odds ratios indicated that the strongest predictors of lane crossing were extreme acute sleepiness, driving long distances, shorter sleep durations in the past week and working night shift. Interventions aimed at reducing the level of sleepiness prior to commuting were suggested.
